# Heat shock protein-90-alpha, a prolactin-STAT5 target gene identified in breast cancer cells, is involved in apoptosis regulation

**DOI:** 10.1186/bcr2193

**Published:** 2008-11-13

**Authors:** Christian Perotti, Ruixuan Liu, Christine T Parusel, Nadine Böcher, Jörg Schultz, Peer Bork, Edith Pfitzner, Bernd Groner, Carrie S Shemanko

**Affiliations:** 1 Department of Biological Sciences, University of Calgary, 2500 University Drive NW, Calgary, AB, T2N 1N4, Canada; 2 Georg-Speyer-Haus, Institute for Biomedical Research, Paul Ehrlich Strasse, 42-44, D-60596, Frankfurt am Main, Germany; 3 European Molecular Biology Laboratories, Meyerhofstrasse 1, 69012 Heidelberg, Germany

## Abstract

**Introduction:**

The prolactin-Janus-kinase-2-signal transducer and activator of transcription-5 (JAK2-STAT5) pathway is essential for the development and functional differentiation of the mammary gland. The pathway also has important roles in mammary tumourigenesis. Prolactin regulated target genes are not yet well defined in tumour cells, and we undertook, to the best of our knowledge, the first large genetic screen of breast cancer cells treated with or without exogenous prolactin. We hypothesise that the identification of these genes should yield insights into the mechanisms by which prolactin participates in cancer formation or progression, and possibly how it regulates normal mammary gland development.

**Methods:**

We used subtractive hybridisation to identify a number of prolactin-regulated genes in the human mammary carcinoma cell line SKBR3. Northern blotting analysis and luciferase assays identified the gene encoding heat shock protein 90-alpha (*HSP90A*) as a prolactin-JAK2-STAT5 target gene, whose function was characterised using apoptosis assays.

**Results:**

We identified a number of new prolactin-regulated genes in breast cancer cells. Focusing on *HSP90A*, we determined that prolactin increased *HSP90A *mRNA in cancerous human breast SKBR3 cells and that STAT5B preferentially activated the *HSP90A *promoter in reporter gene assays. Both prolactin and its downstream protein effector, HSP90α, promote survival, as shown by apoptosis assays and by the addition of the HSP90 inhibitor, 17-allylamino-17-demethoxygeldanamycin (17-AAG), in both untransformed HC11 mammary epithelial cells and SKBR3 breast cancer cells. The constitutive expression of *HSP90A*, however, sensitised differentiated HC11 cells to starvation-induced wild-type p53-independent apoptosis. Interestingly, in SKBR3 breast cancer cells, HSP90α promoted survival in the presence of serum but appeared to have little effect during starvation.

**Conclusions:**

In addition to identifying new prolactin-regulated genes in breast cancer cells, we found that prolactin-JAK2-STAT5 induces expression of the *HSP90A *gene, which encodes the master chaperone of cancer. This identifies one mechanism by which prolactin contributes to breast cancer. Increased expression of *HSP90A *in breast cancer is correlated with increased cell survival and poor prognosis and HSP90α inhibitors are being tested in clinical trials as a breast cancer treatment. Our results also indicate that HSP90α promotes survival depending on the cellular conditions and state of cellular transformation.

## Introduction

The proliferation and functional differentiation of mammary epithelial cells is highly dependent on the action of prolactin [1]. These effects of prolactin are mainly mediated through the prolactin receptor Janus kinase-2-signal transducers and activators of transcription-5 (JAK2-STAT5) pathway, and results in mammary epithelial cell proliferation and the differentiation of alveolar cells during pregnancy. On weaning, a large proportion of these alveolar cells die off in a massive wave of apoptosis and tissue remodelling [2]. Generally speaking, prolactin and STAT5 are thought to induce genes for survival in the differentiated cells, while STAT3 is thought to induce genes required for apoptosis [3].

Prolactin signalling has also been implicated in mammary and breast cancer, including invasive [4] and non-invasive breast cancer [5-7]. The transgenic expression of prolactin results in increased tumour formation in mice [8-10]. Crossing prolactin-deficient mice with oncogenic polyoma middle-T antigen transgenic mice demonstrated that prolactin decreased the latency of tumour formation and increased tumour growth [11]. Using a cross with SV-40T oncogene transgenics and prolactin receptor null mice, and transplant of the epithelium to endocrine normal mice, the prolactin receptor was demonstrated to increase neoplasia and positively impact the transition to invasive carcinoma [12].

In humans, high serum prolactin levels increase the risk of breast cancer for women [4,7,13]. Although expression of the prolactin receptor is more often found in oestrogen receptor-positive breast tumours, which tend to have a better prognosis, it is also found in many oestrogen receptor-negative breast tumours [14]. The gene encoding the prolactin receptor is also highly expressed in a subset of breast tumours with poor prognosis and is part of a set of prognostic gene markers [15]. Prolactin is not only secreted by the pituitary gland, but is also produced locally in the majority of breast tumours and is thought to act in an autocrine and/or paracrine fashion [6]. Although prolactin can transduce signals through multiple pathways, the activation of STAT family members, which are downstream of prolactin and other growth factors has also been implicated in tumourigenesis [16,17]. Prolactin-regulated target genes are not yet well defined in breast cancer cells. Only recently have large-scale attempts at identifying prolactin target genes been performed using the mammary gland and these have been limited to normal mammary epithelial cells [18-24].

The heat shock protein 90-alpha (HSP90α) protein is referred to as the cancer chaperone [25,26], a molecular chaperone of proteins involved in essential signal transduction pathways regulating proliferation, differentiation, apoptosis, angiogenesis, metastasis, oncogenesis [27-29], genetic variation [30,31], invasion [32] and cellular transformation [33]. It is distinct from HSP90β [34], a protein encoded by a related gene, which is constitutively expressed. HSP90α gene expression is elevated in breast cancers [35-37] and is correlated with decreased patient survival [35-37]. HSP90 inhibitors bind specifically and preferentially to HSP90α and β in cancerous cells versus normal cells [38]. Inhibition of HSP90 results in the proteosomal degradation of many HSP90 client proteins. Client proteins include erythroblastic leukaemia viral oncogene homolog 2 (ERBB2) and AKT/protein kinase B [39] and their loss results in apoptosis. HSP90 inhibitors, such as geldanamycin derivatives like 17-allylamino-17-demethoxygeldanamycin (17-AAG), have entered clinical trials for the treatment of breast and prostate cancer and melanoma [27,40,41].

Prolactin-regulated target genes in breast cancer cells are likely to also function in normal cells, but under appropriate regulation. We hypothesised that prolactin-regulated genes in breast cancer cells would also have important functions in non-transformed mammary epithelial cells and may potentially contribute to cancer progression when deregulated. Using a subtractive hybridisation approach, we identified a number of cancer-related genes whose expression is modified by the addition of prolactin in the human breast adenocarcinoma cell line, SKBR3. We specifically identified the therapeutically important gene, *HSP90A *as a STAT5 regulated target gene. *HSP90A *appears to regulate survival differentially depending on the cellular levels of its protein product HSP90α, the presence of survival factors and the status of cellular transformation.

## Materials and methods

### Antibodies

Polyclonal rabbit antibodies against HSP90α were acquired from StressGen (Victoria, BC, Canada), and rabbit anti-STAT5B from Santa Cruz Biotechnology (Santa Cruz, CA, USA). Mouse anti-phospho-histone 2A.X (Ser139) clone JBW301 and anti-GRB2 antibodies (Upstate, Charlottesville, USA) and mouse anti-GRB2 (BD Biosciences, Ontario, Canada) were also acquired. GRB2 was used as a loading control [42].

### Plasmids

The *HSP90A*-luciferase reporter gene contains about 1.8 kb of promoter sequences (Xho I-Hind III) of the human *HSP90A *gene cloned into pLux F3 (KS89 α XL Lux). Expression constructs for the β-galactosidase gene, STAT5A and STAT5B [43], STAT1 and STAT3 (pME18S) and prolactin-receptor [44] have been described. The DNA encoding human *HSP90A *(2199 bp, accession number [GenBank:X15183]) was amplified by PCR from cDNA prepared from SKBR3 cells (SMART cDNA synthesis kit, Clontech Laboratories, Mountain View, CA, USA). The resulting DNA was cut with Bam HI and Not I (New England Biolabs, Ipswich, MA, USA) (adaptors on the primers), subcloned into pcDNA 3.1/Zeo(+) behind the constitutively active cytomegalovirus immediate early promoter (CMV) and the expected sequence verified.

### Cell culture and cell lines

SKBR3 cells, a human breast cancer cell line, were grown in Dulbecco's Modified Eagle Medium with L-glutamine and 10% fetal bovine serum. Undifferentiated HC11 cells, a mouse mammary epithelial cell line [45], were cultured in RPMI with fetal bovine serum and maintained in 0.01 μg/ml epidermal growth factor (EGF) and 5 μg/ml insulin. Confluent cells became competent to respond to lactogenic hormones after incubation in 0.01 μg/ml EGF for one to four days and then 5 μg/ml insulin and 1 × 10^-7 ^M dexamethasone for one day (option if treating with EGF for only one day). Differentiation was then induced (for three days if insulin and dexamethasone pre-treatment was used, or four days if not) with 1 × 10^-7 ^M dexamethasone, 5 μg/ml insulin and 5 μg/ml prolactin. Undifferentiated HC11 cells were transfected with *HSP90A*-pcDNA 3.1/Zeo(+) (HC11-HSP90α) or the empty vector pcDNA3.1/Zeo(+) (HC11-EV) using Lipofectamine 2000 (catalogue 11668-019, Invitrogen Corporation, Carlsbad, California, USA) according to the manufacturer's instructions. Transfected cells were selected as a pool with zeocin treatment and constitutive expression was verified by both northern and western blotting.

### Subtractive hybridisation libraries

RNA was prepared (RNeasy, Clontech, Heidelberg, Germany) from SKBR3 cells seeded at 1 × 10^6 ^and 2 × 10^6 ^cells per 15 cm plate, starved of fetal bovine serum the following day for 16 hours and then treated with or without 5 μg/ml prolactin for 60 minutes in the presence of 10 nM cycloheximide. The subtraction hybridisation libraries were prepared using the SMART PCR cDNA Synthesis Kit and the PCR Select Subtractive Hybridization kit (Clontech, Heidelberg, Germany), and probed using the PCR Select Differential Screening kit (Clontech, Heidelberg, Germany). Positive clones were sequenced to obtain their identity.

### Northern blotting

Total RNA was extracted using peqGOLD Trifast (peqLab, Erlangen, Germany) and resolved on a formaldehyde agarose gel. The blot was blocked using ExpressHyb (Clontech, Heidelberg, Germany), and hybridised with radioactively labelled probes (Strip-EZ DNA, Ambion, AMS Biotechnology Ltd). A DNA probe encoding exon 3 of the human *CIS *gene [46] was amplified using the forward primer 5'-GCT GGT ATT GGG GTT CC-3' and the reverse primer 5'-TGA GGG CTC TGT ACA TGA AAG-3' The fragments were gel purified (Qiaquick Gel Extraction Kit, Qiagen GmbH, Hilden, Germany).

### Electrophoretic mobility shift assays

Electrophoretic mobility shift assays using a radioactively labelled fragment of the bovine β-casein promoter were performed as described [47]. Essentially the double strand STAT responsive element from the β-casein promoter was prepared and radioactively labelled before incubation with protein extracts. The complexes were resolved on a non-reducing gel and autoradiographed.

### Transfection and luciferase assays

Transfection using calcium chloride and luciferase assays using HeLa cells was performed as described [48] using overnight treatments with 5 μg/ml prolactin. A β-galactosidase gene was included to compare transfection efficiencies in individual experiments.

### Western blotting

Soluble protein extracts were prepared in a buffer containing 1% Nonidet-P-40, 50 mM Tris pH 7.5, 5 mM ethylene glycol tetraacetic acid and 200 mM sodium chloride, with freshly added protease and phosphatase inhibitors: 1 mM sodium vanadate, 20 μM phenylarsine oxide, 1 μg/ml leupeptin, 0.5 μg/ml aprotinin, 100 μM phenylmethylsulphonyl fluoride and 1 mM DTT. After protein concentrations were measured with the Bio-Rad Assay, 50 μg of each lysate was resolved by 15% SDS-PAGE and then transferred to Hybond-P PVDF transfer membrane (catalogue RPN303F, Amersham, GE Healthcare, Baie d'Urfé, Québec, Canada). The membrane was blocked in 5% non-fat milk in tris-buffered saline with 0.05% Tween 20 and incubated with 1 μg/ml of primary antibody mouse anti-phospho-histone H2A.X (Ser139), clone JBW301 (Upstate, Millipore, Billerica, MA, USA) followed with horseradish peroxidase (HRP)-conjugated goat anti-mouse secondary antibody. The signal was developed by solutions prepared with 250 mM luminal solution, 90 mM p-coumaric solution, 1 M Tris pH 8.5 and 30% hydrogen peroxide.

### Apoptosis assay

HC11 cells transfected with either the empty vector HC11-EV or with the HSP90α expression construct (HC11-HSP90α) were plated at 130,000 cells/well of a 96-well plate and differentiated as above, then starved by the absence of serum and hormones. SKBR3 cells were plated at 10,000 cells/well. The presence of mono- and oligo-nucleosomes in the cytoplasm were qualitatively measured using the Cell Death Detection Elisa Plus Kit (Roche, Mississauga, ON, Canada). Essentially, protein extracts were incubated with anti-DNA (HRP-coupled) and anti-histone (biotin coupled) antibodies, before incubation in streptavidin-coated 96-well plates. Colorimetric detection was performed at the absorbance wavelength of 405 to 490 nm.

## Results

### SKBR3 human breast carcinoma cells are responsive to prolactin through STAT5-mediated gene transcription

**Figure 1:**
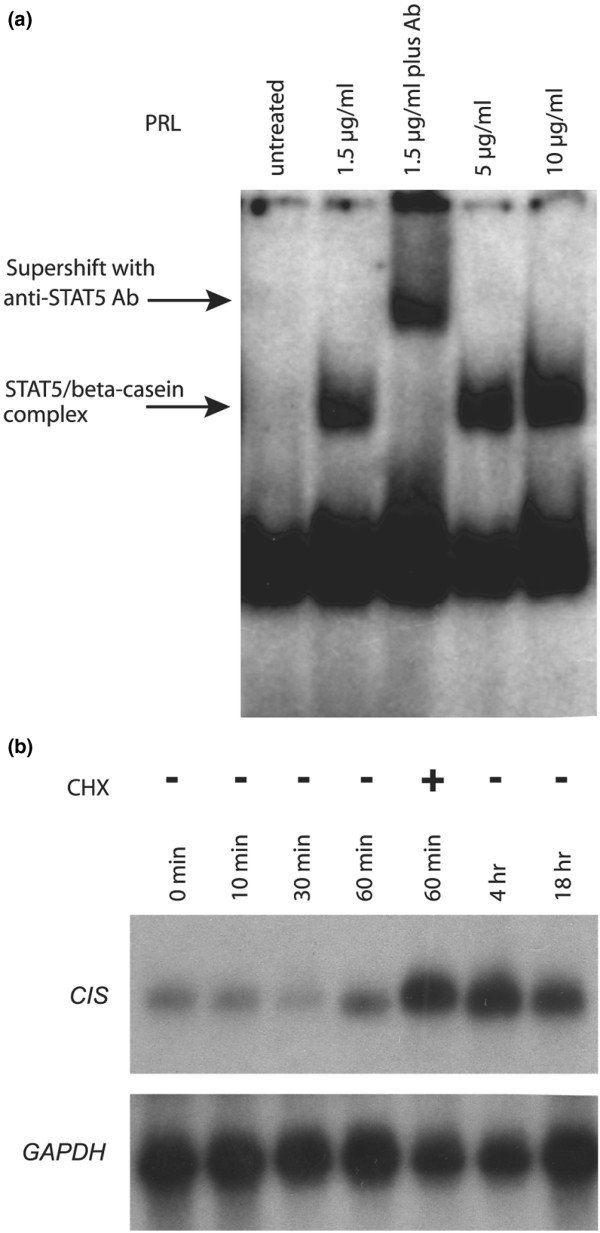
**Prolactin-STAT5-mediated signal transduction in SKBR3 cells**. **(a) **Electrophoretic mobility shift assays were carried out with a radioactively labelled β-casein gene promoter probe and protein extracts of SKBR3 cells treated for one hour with the indicated amounts of prolactin (PRL). The arrows indicate the positions of the signal transducers and activators of transcription (STAT5) DNA complex and of a supershifted complex formed in the presence of a STAT5 specific antibody (Ab). **(b) **SKBR3 cells were seeded one day, the next day starved of serum overnight and the following day were stimulated (or not) with 5 μg/ml prolactin for the times indicated in the absence of cycloheximide (CHX) unless indicated. In each lane 20 μg of RNA were applied. The northern blot was first hybridised with labelled DNA encoding cytokine inducible SH2 containing protein *(CIS) *and then rehybridised with a glyceraldehyde 3-phosphate dehydrogenase (*GAPDH*) specific probe.

To investigate the role of prolactin in breast cancer, we set out to identify prolactin responsive genes in the breast cancer cell line, SKBR3. We first examined the prolactin-based activation of STAT5 and the induction kinetics of previously identified STAT5-dependent genes. SKBR3 cells were treated with different doses of prolactin and the activated DNA-binding form of STAT5 was visualised in electrophoretic mobility shift assays (Figure 1a). The experiment shows that prolactin is able to activate STAT5 in SKBR3 cells in a dose-dependent manner. A STAT5 specific antibody was used to confirm the specificity of the protein-DNA complex (Figure 1a). Prolactin stimulation of 60 minutes resulted in activation and binding of STAT5 to DNA response elements present in the β-casein promoter. Of note is the lack of STAT5 activation in the absence of prolactin stimulation. SKBR3 cells have previously been shown to express the prolactin gene [49], but perhaps the endogenous levels of prolactin are not sufficient to induce activation of the JAK2-STAT5 pathway.

We next followed the kinetics of cytokine-inducible-SH2-containing protein (*CIS*) mRNA induction as a function of prolactin treatment of SKBR3 cells. The *CIS *gene [50] is a known target of the JAK2-STAT5 pathway activated by interleukin-2 or erythropoietin in lymphoid cells and is important for feedback inhibition [51,52]. The amount of *CIS *mRNA increased within 60 minutes of treatment and was further enhanced in the presence of cycloheximide, an inhibitor of protein synthesis (Figure 1b). The level of *CIS *mRNA reached a maximum at four hours and remained high for at least 18 hours. This confirmed that SKBR3 cells are appropriate to study the early induction of prolactin-JAK2-STAT5-regulated genes and that a time point of 60 minutes would result in the production of early target genes.

### Prolactin-regulated genes in SKBR3 breast cancer cells

To identify additional genes regulated by prolactin in SKBR3 cells, we prepared subtraction hybridisation libraries. Based on the above observations, SKBR3 cells were treated for 60 minutes with 5 μg/ml prolactin and the RNA was used for preparation of the subtractive hybridisation libraries. Cycloheximide was added to cell preparations, both untreated and treated with prolactin in order to avoid identification of secondary targets. We prepared subtraction libraries for the genes differentially expressed on prolactin treatment (forward) and for genes expressed in the absence of prolactin (reverse). The two libraries were used in the differential screen for prolactin-regulated genes. About 1200 gene fragments were screened from the forward library and 770 from the reverse library. Genes were screened using Southern blotting in batches of 100 genes using the forward and reverse libraries as probes. The genes with the most intense differential signal on each blot, as measured using a Biorad phosphoimager (Bio-Rad, Munich, Germany), were selected for sequencing. Seventy-two positive clones selected on the basis of high expression in the forward (Table 1) or reverse libraries (Table 2) were sequenced and identified. Some genes were observed more than once (Tables 1 and 2), also indicating high differential expression. Of the 51 genes represented, 19 genes with the highest expression levels were rescreened in dot-blots using both the unsubtracted probes (cDNA) and subtracted probes and the expression patterns of 16 of the 19 genes were confirmed (86%) (Tables 1 and 2).

**Table 1:** Forward library – Genes upregulated in the presence of prolactin

**Confirmed**	**Observed**	**Function**	**Accession number**	**Protein name**
		**RNA-related functions**		
		
		mRNA splicing	[Swiss-Prot:Q8IYB3]	Serine/arginine-related nuclear matrix protein
		mRNA transport	[Swiss-Prot:Q8N1F7]	KIAA0095 protein, Nup93
	2	RNA-binding protein	[Swiss-Prot:P09651]	Heterogeneous nuclear ribonucleoprotein A1

		**Chaperone**		
		
*	5	Heat shock	[GenBank:BAA13431] [Swiss-Prot:O75322]	Heat shock protein 90 alpha and beta
	2	Heat shock	[GenBank:AAD11466] [GenBank:AAD21817] [GenBank:AAD21815] [GenBank:AAD21816] [Swiss-Prot:P34931]	Heat shock 70 KD protein 1 and 2 and heat shock 70 KD protein 1-like
		Part of the TCP1 complex	[GenBank:CAG33352]	TCP-1-beta, CCT-beta
		ER-resident chaperone	[Swiss-Prot:P06761]	GRP78, BiP

		**Protein synthesis**		
		
*	5	Protein synthesis	[Swiss-Prot:Q14222]	Elongation factor-1 alpha
		Upregulated in metastasis	[Swiss-Prot:Q9NQ50]	39S ribosomal protein L40, mitochondrial precursor, L40mt, MRP-40

		**Electron transport**		
		
*	2	Oxidative phosphorylation	[GenBank:AAD13930]	Mitochondrial hinge protein, OXPHOS system complex III mitochondrial subunit
		Mitochondrial electron transport chain	[Swiss-Prot:Q15070]	inner membrane protein OXA1L, mitochondrial precursor
		Mitochondrial electron transport chain	[GenBank:BAA77673]	NADH dehydrogenase subunit 4

		**Transport**		
		
		ER-to-Golgi transport	[Swiss-Prot:O75935]	Dynactin subunit 3, dynactin complex subunit 22 kDa subunit, p22

		**Proteasome**		
		
		Regulatory subunit of proteasome function	[Swiss-Prot:Q13041]	26S proteasome regulatory subunit S2, p97, tumour necrosis factor type 1 receptor-associated protein 2
		Proteasome	[Swiss-Prot:P25789]	Proteasome component C9, macropain subunit C9

		**Ubiquitination**		
		
		Heterodimerising with cullin-1 to catalyse ubiquitin polymerisation	[GenBank:AAD29715]	Ring box protein 1

		**Survival**		
		
*		Survival	[GenBank:AAB87479]	TEGT protein, BAX inhibitor 1
*	2	Caspase-9 inhibition	[Swiss-Prot:O00165]	HA1-associating protein Hs1-binding protein

		**Miscellaneous**		
		
		Activity in milk	[Swiss-Prot:P07195]	L-lactate dehydrogenase H chain LDH-B
	2	ECM-receptor interaction	[GenBank:BAA76814]	KIAA0970 protein, fibronectin type III domain containing 3A
		Toll-like receptor-mediated interferon response	[GenBank:AAD34590]	T2K protein kinase homolog (mouse)
*	2	Iron storage	[Swiss-Prot:P02794]	Ferritin heavy chain, ferritin H subunit
		Endosome location	[Swiss-Prot:Q99805]	Transmembrane 9 superfamily protein member 2 precursor, p76
		Regulation of nonmuscle myosin II	[Swiss-Prot:O14950]	Myosin regulatory light chain
		nucleic acid synthesis	[Swiss-Prot:P30085]	UMP-CMP kinase 1, cytidylate kinase
		Retaining proteins in the ER	[Swiss-Prot:Q08013]	TRAP gamma, SSR-gamma
*		Peptide-modifying enzyme component	[Gi:2894085]	P40 mRNA for lanthionine synthetase C-like protein 1

**Prolactin-regulated genes**. cDNA fragments identified preferentially in the forward library (presence of prolactin) was sequenced. Sequences that were identical to those that encoded human proteins are presented using the protein accession number. Genes that were identified more than once are indicated as the number of times observed. Genes that were confirmed by a second screen are marked with an asterisk.

BAX = Bcl2-associated X protein; BiP = binding protein; CCT-beta = chaperonin-containing TCP-1 complex; UMP-CMP = citidine monophosphate; ER = endoplasmic reticulum; GRP78 = 78 KD glucose-regulated protein precursor; NADH = nicotinamide adenine dinucleotide plus hydrogen; Nup93= nuclear pore complex protein; NF45 = nuclear factor of activated T-cells 45 kDa; OXA1L = oxidase assembly 1-like protein; OXPHOS = oxidative phosphorylation; TCP-1-beta = T-complex protein 1, beta subunit; TEGT = testis enhanced gene transcript; TRAP gamma = translocon-associated protein, gamma subunit; SSR-gamma = signal sequence receptor gamma subunit.

**Table 2:** Reverse library – Genes upregulation in the absence of prolactin

**Confirmed**	**Observed**	**Function**	**Accession number**	**Protein name**
		**RNA-related functions**		
		
		Splicing factor	[Swiss-Prot:O75533]	Splicing factor 3B subunit 1, spliceosome-associated protein 155
		Translational regulation	[Swiss-Prot:Q8TB72]	Pumilio homolog 2, pumilio-2, KIAA0235 fragment

		**Chaperone**		
		
		Chaperone	[Swiss-Prot:O60925]	Prefoldin subunit 1

		**Protein synthesis**		
		
*		Activates the trk oncogene	[Swiss-Prot:P62424]	60S ribosomal protein L7A, surfeit locus protein 3, PLA-X polypeptide

		**Electron transport**		
		
		Electron transport	[GenBank:AAC25442]	NADH dehydrogenase subunit 2 homo sapiens
		Selenium metabolism and protection oxidative stress	[Swiss-Prot:Q99475]	Km-102-derived reductase like factor, thioredoxin reductase
		Electron transport	[RefSeq:NP_055217] + [GenBank:EAW62308] [GenBank:AAH90048] [GenBank:AAH01390] [Swiss-Prot:O14949]	Ubiquinol-cytochrome c reductase complex ubiquinone binding protein QP-C, complex III subunit VII

		**Transport**		
		
*		Vesicle trafficking protein transport	[GenBank:BAA07558]	Hypothetical protein KIAA0079, HA3543, SEC24-related protein C
*		Protein transport	[GenBank:CAI15005]	Coatomer alpha subunit, Alpha-COP

		**Proteasome**		
		
*		Proteasome	[Swiss-Prot:P25788]	Proteasome component C8, macropain subunit C8, proteasome subunit alpha type 3 multicatalytic endopeptidase complex subunit C8

		**Ubiquitination**		
		
		Deubiquitinating enzyme tumour suppressor	[GenBank:BAA74872]	KIAA0849 protein, CYLD gene
		Ubiquitin-specific protease cysteine proteases	[GenBank:AAH64516] [GenBank:NP_005144] [Swiss-Prot:Q14694] [GenBank:ABM86690] [GenBank:ABM83479]	KIAA0190 ubiquitin specific peptidase 10
*		Ubiquitin cycle	[GI:4929720] [GenBank:NP_057490] [Swiss-Prot:Q9Y3C8] [GenBank:AF151884]	Ubiquitin-fold modifier conjugating enzyme 1

		**Transcription factors**		
		
*		Transcription factor	[Swiss-Prot:Q12905]	Interleukin enhancer-binding factor 2, NF45 protein
		Transcription factor complex, RNA binding	[GenBank:CAA10029]	NS1-binding protein

		**Miscellaneous**		
		
*	2	N-oligosaccharyl transferase complex	[GenBank:CAB41763]	DJ343K2.2.1, ribophorin II isoform 1
		Unknown	[Swiss-Prot:O95801]	Tetratricopeptide repeat protein 4
		Kinase	[GenBank:BAA76815]	KIAA0971 protein, FAST kinase domains 2
		Mitochondrial fusion	[Swiss-Prot:O95140] [GenBank:AAD02058]	MFN2, KIAA0214 protein, CPRP1
		Calcium binding	[Swiss-Prot:P62158]	Calmodulin
		Glycolytic and gluconeogenesis pathways Second product-transcription factor	[Swiss-Prot:P06733]	Alpha-enolase, 2-phospho-D-glycerate hydro-lyase, enolase 1, MBP-1, plasminogen-binding protein
	2	Palmitoyl-(protein) hydrolase activity	[PIR:I58097]	Palmitoyl protein thioesterase precursor, EC 3.1.2.22
*		Iron ion transport	[GenBank:gi:37432]	Transferrin receptor, p90, CD71
*	2	Unknown OR Wnt signalling pathway	[GenBank:gi:1167502] [GenBank:gi:1524104]	Hypothetical protein TI-227H wnt 13

**Prolactin-regulated genes**. cDNA fragments identified preferentially in the reverse libraries (absence of prolactin) were sequenced. Sequences that were identical to those that encoded human proteins are presented using the protein accession number. Genes that were identified more than once are indicated as the number of times observed. Genes that were confirmed by a second screen are marked with an asterisk.

Alpha-COP = alpha coat protein; CPRP1 = caprine prolactin-related protein-1; CYLD = cylindromatosis; FAST = Fas-activated serine/threonine; MFN2 = mitofusin-2, transmembrane GTPase; MBP-1 = C-myc promoter-binding protein; NADH = nicotinamide adenine dinucleotide plus hydrogen; NS1 = non-structural 1 protein; NF45 = nuclear factor of activated T-cells 45 kDa; wnt 13 = wingless-type MMTV (mouse mammary tumour virus) integration site family, member 2B.

Although there were no pre-existing large studies of prolactin-regulated genes in breast cancer cells, we compared our results with other prolactin-related studies. Of note, 11 of the genes we identified overlapped with those previously identified as downstream targets of the prolactin pathway. In one study, prolactin gene targets were identified by gene array using regenerated mammary glands from prolactin-receptor-/- and cyclin D-/- mammary epithelial cell transplants. Cyclin D1 was thought to represent a secondary target of prolactin, and therefore genes identified in this screen would represent effectors downstream of prolactin and upstream of cyclin D1 [18]. Although the transplants were nontransformed cells, unlike SKBR3 cells, we noted some similarities. Genes that were identical in this study and our report include the genes encoding the mouse ferritin heavy chain gene, HSPs 70, 71 and 84 (*HSP90B*). We also found overlap with prolactin-regulated genes identified in the rat Nb2-11c lymphoma cell line [53], including *HSP70 *and *HSP86 *(*HSP90A*). Genes found to be similar (either functionally related or different subunits of a complex) between these two studies and ours include Sec 23 [18] and Sec 22 [53] (similar to Sec 24 in our study), elongation factor 2 [18,53] (similar to elongation factor 1 alpha), lactate dehydrogenase 1 A chain [18] (similar to lactate dehydrogenase H chain), myosin heavy chain (similar to myosin regulatory light chain), T-complex protein 1 e and h subunits [53] (similar to T-complex b subunit). The degree of overlap with the prolactin-regulated genes of these two studies is comparable with the level previously described between other prolactin target gene studies [19]. The diversity of the gene lists found in each study is thought to derive from the differences between experimental conditions, cell types and methods.

### *HSP90A *is a prolactin-induced gene in SKBR3 human breast cancer cells

The gene *HSP90A *was identified five times in the initial group selected for sequencing; and based on its high representation, differential expression and its function in cancer cells, was used for further analysis.

**Figure 2:**
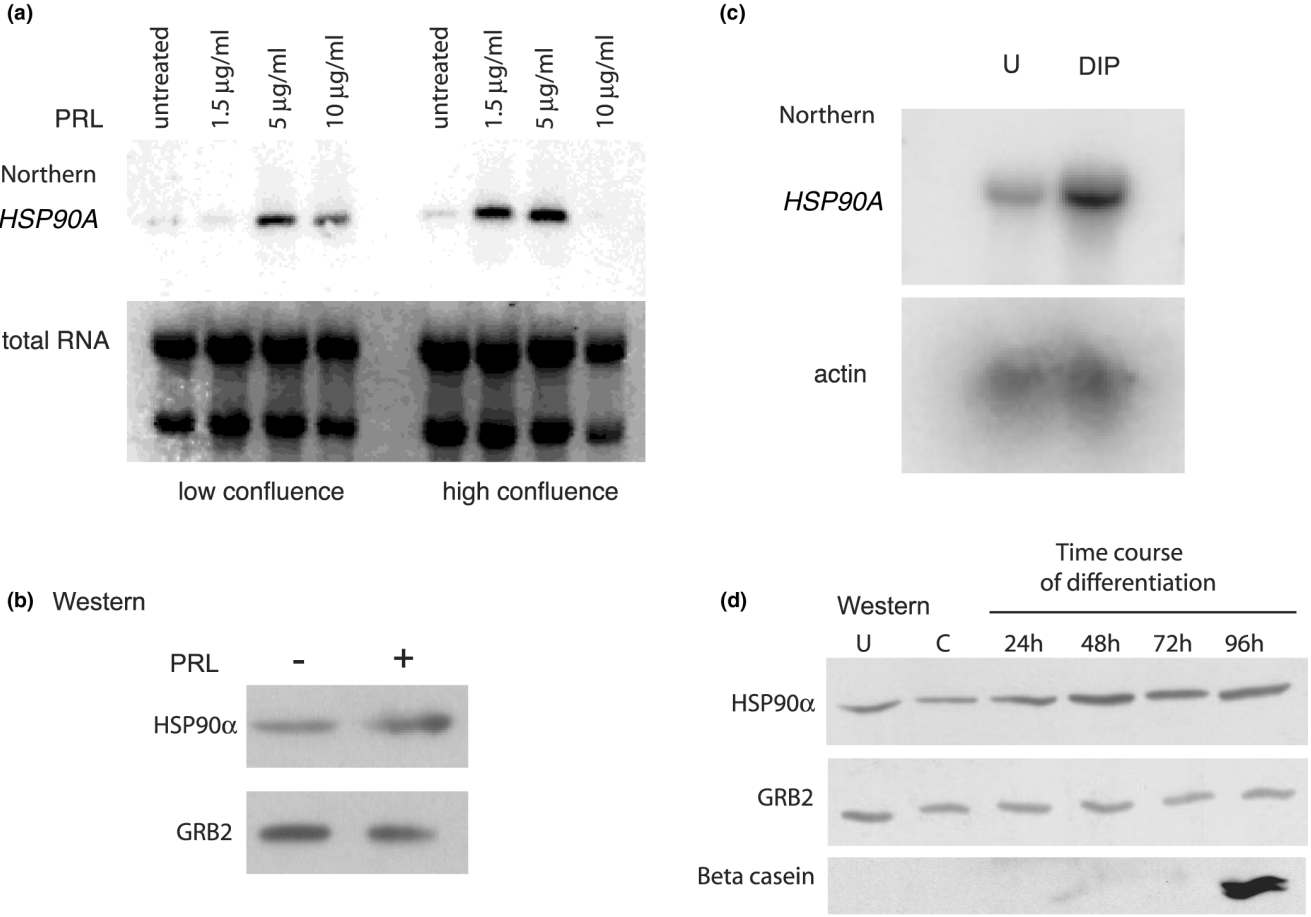
**HSP90α is a prolactin regulated target gene**. **(a) **Dose response of the heat shock protein 90 alpha (*HSP90A*) gene to prolactin in SKBR3 cells assessed by northern analysis. SKBR3 cells were seeded to achieve low and higher confluence before treatment for one hour with the indicated doses of prolactin (PRL). Total RNA was transferred to a nylon membrane, and the blot probed with the *HSP90A *gene fragment. **(b) **Western blot analysis: HSP90α protein is increased about two-fold in SKBR3 cells in response to 5 ug/mL prolactin. **(c) **Northern analysis: HSP90α mRNA is increased about four-fold in HC11 cells in response to a one hour treatment of lactogenic hormones, prolactin, dexamethasone and insulin (DIP) compared with undifferentiated (U) HC11 cells treated with epidermal growth factor and insulin. **(d) **Western blot of HSP90α protein extracts from undifferentiated, competent (C) HC11 cells or after DIP treatment for the time indicated. Production of beta-casein is observed after 96 hours when the cells are fully differentiated. Longer exposures show a small amount of beta-casein after 72 hours.

We used northern blotting analysis to independently verify the induction of the *HSP90A *gene in SKBR3 cells by prolactin. We plated cells at two different confluences (about 40% and 60% confluent on the day of stimulation) as was performed for the preparation of the library. Both populations responded to 60 minutes of prolactin treatment with the increase in *HSP90A *mRNA, but the dose response of *HSP90A *induction in the two-cell population was distinguishable. Cells at the higher confluence exhibited a maximal response at 1.5 μg/ml, whereas cell at lower confluence required 5 μg/ml for maximal induction (Figure 2a). Higher concentrations of prolactin reduce the maximal response. The observed lower level of total RNA in high confluence cells treated with 10 μg/ml prolactin, and potentially the inhibitory presence of *CIS*, may explain the reduction in signal observed in higher confluence cells at 10 μg/ml. Another alternative is that the high concentration of prolactin induced a refractory state of prolactin signal transduction [54]. These results confirm that the *HSP90A *gene is a prolactin-regulated gene in the human mammary carcinoma cell line, SKBR3.

We then investigated whether there was an increase in the encoded protein HSP90α in prolactin-treated SKBR3 cells. SKBR3 cells were cultured in the absence or presence of prolactin and protein extracts were resolved by SDS-PAGE. Western blotting using antibodies directed against HSP90α indicated that there is a two-fold increase in the amount of HSP90α protein in prolactin treated cells (Figure 2b). Prolactin therefore induces both the expression of *HSP90A *mRNA and increases HSP90α protein in breast cancer cells.

We also investigated the response of the *HSP90A *gene in mouse mammary epithelial HC11 cells. HC11 cells exist in an undifferentiated state until competent cells are stimulated appropriately with lactogenic hormones, dexamethasone, insulin and prolactin, to differentiate and produce milk proteins [55]. We compared the levels of *HSP90A *mRNA in HC11 cells in their undifferentiated state and after a one-hour treatment of competent cells with lactogenic hormones, including prolactin (Figure 2c), simulating the time point used in SKBR3 cells. Lactogenic hormone induction resulted in a rapid four-fold increase of *HSP90A *mRNA in HC11 cells, as quantified by phosphoimager analysis of northern blots using actin as a loading control. We also observed up to a two-fold increase in HSP90α protein, peaking at 48 hours of lactogenic hormone induction of HC11 cells (Figure 2d). This demonstrated that the *HSP90A *mRNA and protein are elevated during early mammary epithelial cell differentiation in response to lactogenic hormones.

### The promoter of the *HSP90A *gene is preferentially activated by STAT5B

Inspection of the human *HSP90A *gene upstream sequence indicated the presence of at least two potential STAT-binding DNA elements that could bind STAT1, STAT3 or STAT5 [56] (nucleotides 1611-1603 and 1177–1185, [GenBank:U25822]). Although STAT1 and STAT3 have been reported to respond to prolactin, the prolactin signal is mainly conferred through the activation of STAT5A and STAT5B, two highly homologous members of the STAT family [57]. In order to investigate the prolactin responsiveness of the *HSP90A *gene promoter, we conducted reporter assays using a gene construct containing about 1.8 kb of the human *HSP90A *upstream regulatory sequence fused to a luciferase reporter gene. We transfected HeLa cells with expression vectors for the long form of the human prolactin-receptor, the *HSP90A*-luciferase reporter construct and various STATs.

**Figure 3:**
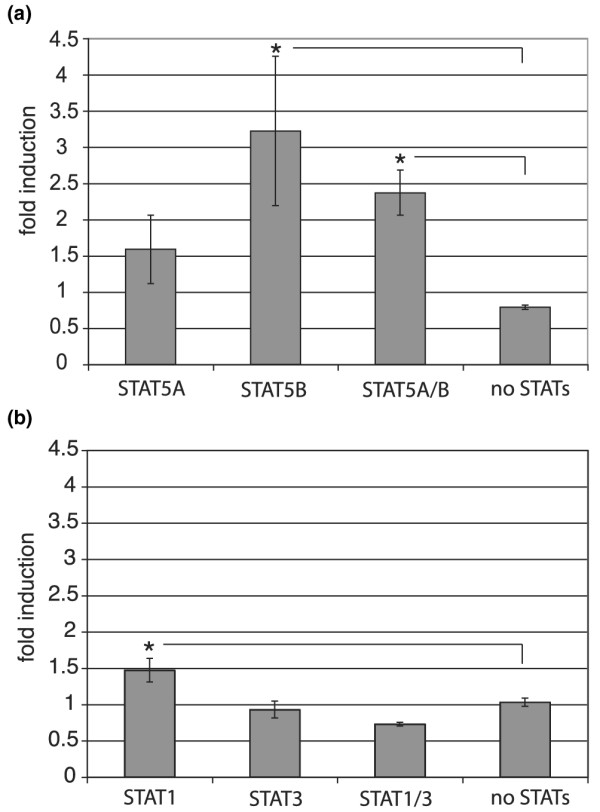
**Prolactin-STAT5 induction of a *HSP90A *promoter-luciferase reporter gene**. HeLa cells were transiently cotransfected with DNA encoding the prolactin-receptor, the indicated signal transducers and activators of transcription (STATs), the heat shock protein 90 alpha (*HSP90A*)-luciferase reporter and β-galactosidase. Luciferase assays were performed 48 hours post transfection and the luciferase activity values normalised with β-galactosidase levels. Fold induction was calculated using the normalised luciferase activity from transfected cells in the absence of prolactin. An asterix indicates that the results are significantly different (p < 0.05 t-test) compared with the sample with no STAT proteins. Each bar represents the average of three to five experiments with standard deviation. **(a) **STAT5A and/or STAT5B. **(b) **STAT1 and/or STAT3.

Prolactin activation of cells transfected with STAT5B, or STAT5A and STAT5B together, caused over a four-fold or an over two-fold increase in luciferase activity, respectively, when compared with cells without exogenous STAT5 expression (Figure 3a). As the induction in the presence of exogenous STAT5A alone was not statistically significant, the significance of the STAT5A/5B result may be due to the presence of STAT5B. The smaller effect of the combination of STAT5A/5B over STAT5B alone may be due to the sequestering of STAT5B through the formation of heterodimers. We also performed reporter assays in both COS-7 as well as SKBR3 cells and obtained similar results with respect to the preferential transcription of the reporter by STAT5B rather than STAT5A (data not shown). In HeLa cells, a small effect of STAT1 and no effect of STAT3 on luciferase activity were observed (Figure 3b). STAT5B is the predominant form of STAT5 in breast tumour cell lines including SKBR3 [58] and most likely contributes to the elevated expression of HSP90α in breast cancer cells. These reporter assays confirm that *HSP90A *is a prolactin-STAT5 regulated target gene.

### Prolactin acts as a survival factor in differentiated HC11 cells

**Figure 4:**
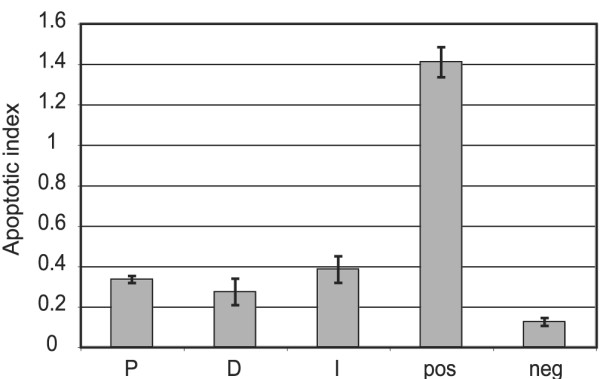
**Prolactin is a survival factor for HC11 cells**. Differentiated HC11 cells were starved of serum and hormones and with specific hormones added back alone as indicated. After 72 hours, cytoplasmic extracts were prepared and analysed by sandwich ELISA for apoptotic mono- and oligo-nuclesomes. All rescue treatments of prolactin (P), dexamethasone (D) or insulin (I) significantly reduced apoptosis caused by starvation of hormones and serum (pos), for example, between prolactin rescue and positive control, p = 0.001 in a t-test. Negative control (neg) are cells in differentiation medium (DIP) without starvation. Each bar represents the average of three experiments with standard deviation.

Following the line of reasoning that prolactin acts as a survival factor in breast cancer cells [59-63] and that STAT5 promotes survival in normal mammary epithelial cells [64,65], we tested whether the addition of prolactin to starved, untransformed mammary epithelial HC11 cells would rescue differentiated cells from apoptosis. HC11 cells were induced to differentiate after they reached confluence by the addition of the lactogenic hormones prolactin, dexamethasone and insulin. Differentiated HC11 cells were then starved of serum and lactogenic hormones with individual hormones returned as indicated for 72 hours, followed by analysis of mono- and oligo-nucleosomes as an indicator of apoptosis (Figure 4). Serum and hormone withdrawal of HC11 cells is known to induce apoptosis [66]. Each of the lactogenic hormones, including prolactin, greatly protects HC11 cells equally well from apoptosis when serum and other lactogenic hormones are removed.

### Constitutive *HSP90A *expression sensitises mammary epithelial cells to apoptosis in starved HC11 cells

We reasoned that HSP90α would have important functions downstream of prolactin not only in cancerous breast cells, but also in untransformed mammary epithelial cells, such as HC11. For this purpose, we created HC11 cell lines that either constitutively expressed the gene for human HSP90α (HC11-HSP90α) or carried the empty vector (HC11-EV). There is a two-fold increase in HSP90α in the HC11-HSP90α line. Given that we showed that prolactin is a survival factor in HC11 cells, we then investigated whether the HC11-HSP90α cells were susceptible to apoptosis induced by the removal of prolactin and other survival factors. We used two independent methods.

**Figure 5:**
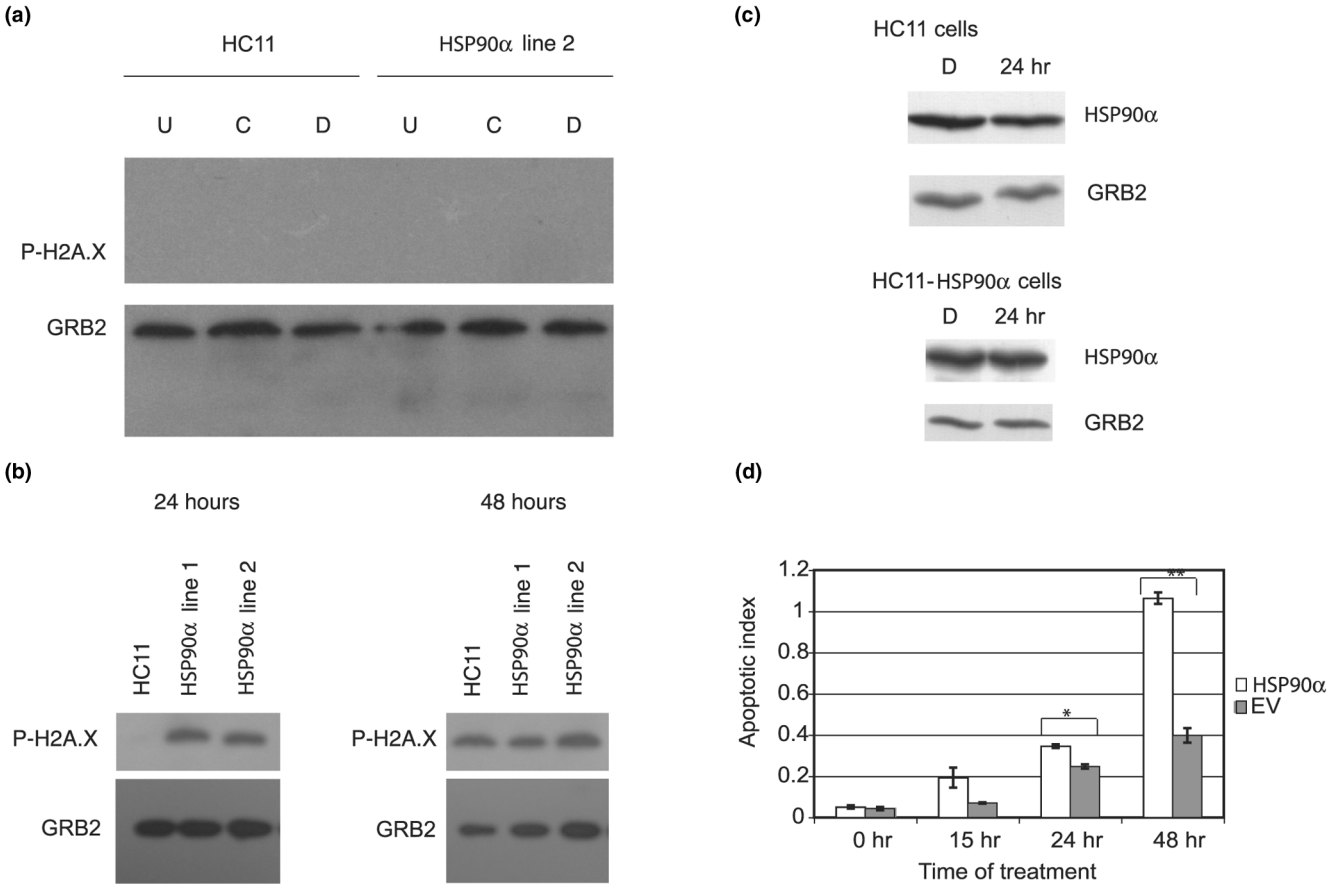
**Constitutive *HSP90A *expression sensitizes cells to apoptosis**. **(a) **Protein extracts were prepared from control parental HC11 or heat shock protein 90 alpha (HSP90α)-HC11 (line 2) cells that were cultured as undifferentiated (U), competent (C) or differentiated (D) cells. **(b, c) **Differentiated parental HC11 and two different pools of HC11-HSP90α cells (lines 1 and/or 2) were induced to differentiate and then starved of lactogenic hormones and serum for the time indicated. Equal amounts of protein were resolved by SDS-PAGE (15%). An antibody was used to detect phosphorylated-histone 2A.X, and an antibody against GRB2 was used as a loading control. **(d) **Differentiated control HC11 and HC11-HSP90α cells (line 2) were starved or not apoptosis was assessed by the relative quantities of mono- and oligo-nucleosomes (apoptotic index). Each bar represents the average of three experiments with standard deviation. t-test, *p = 0.02 at 24 hours, **p = 0.00004 at 48 hours.

First, we used an antibody against phosphorylated-histone 2A.X as a marker of the apoptotic DNA damage [67] that occurs in response to starvation. As expected, there was little to no indication of phosphorylated-histone 2A.X in the undifferentiated, competent or differentiated cells, which are cultured in the presence of serum and hormones (Figure 5a). Differentiated cells were then starved of serum and all lactogenic hormones for up to 48 hours to induce apoptosis. The HC11-HSP90α cell lines were more sensitive to starvation than the parental HC11 cells or cells expressing the empty vector. After 24 hours of serum and hormone withdrawal, phospho-histone 2A.X is easily detected by western blot in the HC11-HSP90α cells, but not as easily in the control HC11 cells (Figure 5b) or cells carrying the empty vector (HC11-EV) (not shown as the response was similar).

We then investigated whether the amount of HSP90α differs between the differentiated and starved cells. When cells are starved for 24 hours, the amount of HSP90α in control cells decreases slightly, but remains more constant in the cells constitutively expressing *HSP90A*, HC11-HSP90α cells (Figure 5c). We can not be certain that the effect on survival we observed (shown in Figure 5b) is due to the differential protein levels between the differentiated and starved cells in the two cell lines, or to the overall two-fold elevated levels of HSP90α in the HC11-HSP90α line. The greater amounts of phospho-histone 2A.X at 24 hours of starvation indicate that constitutive expression of the gene encoding HSP90α increases levels of the marker for DNA damage, phospho-histone 2A.X, and may sensitise the cells to apoptosis.

To confirm that the phosphorylation of histone 2A.X represented events that occur during apoptosis, we also qualitatively assayed the mono- and oligo-nucleosomes generated due to apoptotic DNA nuclease activation. As both HC11-HSP90α cell lines behaved similarly, we used only line two for further characterisation. The HC11 cell lines were treated as shown in Figure 5b, and equal amounts of the cytoplasmic extracts were assessed for the presence of mono- and oligo-nucleosomes. HC11-HSP90α cells clearly had higher levels of nucleosomes than control cells HC11-EV (empty vector) after 24 and 48 hours of starvation, indicating greater levels of apoptosis (Figure 5d). These results support that although prolactin acts as a survival factor in the absence of prolactin or other survival factors, HC11 cells constitutively expressing HSP90α are sensitised to starvation-induced apoptosis.

**Figure 6:**
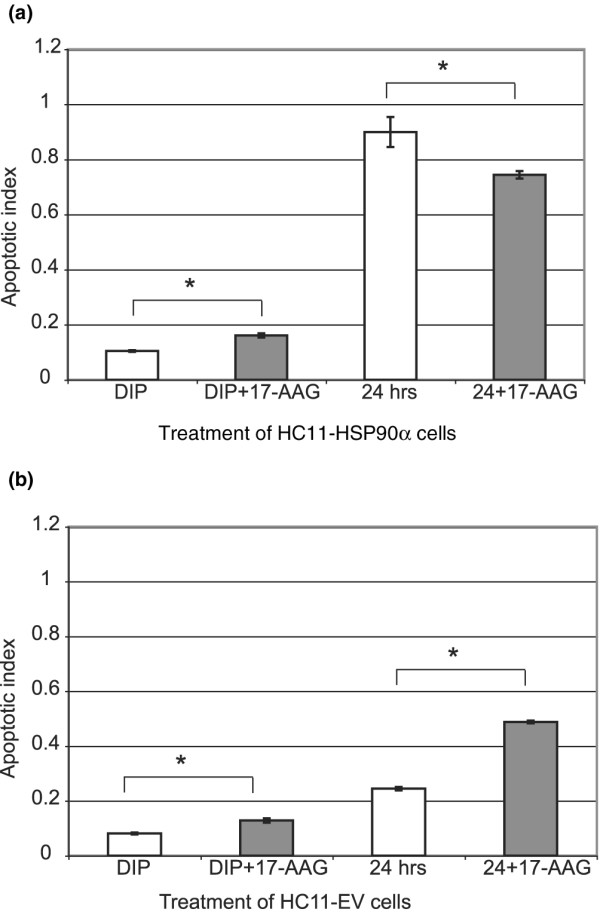
**Inhibition of HSP90α by 17-AAG defines roles for survival as well as apoptosis in HC11 cells**. The presence of mono- and oligo-nucleosomes was measured as an indication of apoptosis (apoptotic index). **(a) **HC11-heat shock protein 90 alpha (HSP90α) cells or **(b) **HC11-EV cells were differentiated and left untreated or treated with 1 μM of 17-allylamino-17-demethoxygeldanamycin (17-AAG) in either differentiation medium (prolactin, dexamethasone and insulin (DIP)) or starvation medium for 24 hours. Comparison of untreated to 17-AAG-treated HC11-HSP90α (t-test *p = 0.003) or HC11-EV (t-test *p = 0.006) cells after differentiation. Comparison of untreated to 17-AAG-treated HC11-HSP90α (t-test *p = 0.04) or HC11-EV (t-test *p = 0.001) cells in the absence of serum and hormones. Each bar represents the average of three experiments with standard deviation. Each cell line was tested independently and so the absolute levels of nucleosomes can be compared only within each panel.

We used the HSP90 inhibitor 17-AAG to confirm our results. First, we confirmed that HSP90α promotes survival in the presence of prolactin and serum. Differentiated HC11-HSP90α (Figure 6a) or HC11-EV cells (Figure 6b) were untreated or treated with 1 μg/mL 17-AAG either in differentiation medium (dexamethasone, insulin and prolactin plus serum) or starvation medium (no serum or hormones) for a total of 24 hours before measuring apoptosis. Consistent with the role of HSP90α promoting survival, inhibition of HSP90 by 17-AAG induced apoptosis in differentiated HC11-HSP90α (Figure 6a) and HC11-EV cells (Figure 6b) in the presence of survival factors such as prolactin and serum. HSP90α also promotes survival in the control HC11-EV cell line in starvation medium, as demonstrated by the increase in apoptosis with the addition of the inhibitor 17-AAG (Figure 6b).

Second, we confirmed that constitutive expression of HSP90α promotes apoptosis in starved cells. Consistent with the effect of constitutive HSP90α expression on the sensitisation of HC11 cells, 17-AAG reduced the starvation-induced apoptosis (Figure 6a). Overall, starvation enhanced the general level of apoptosis in differentiated cells of both cell lines, although the effects of the HSP90 inhibitor were different. Together this confirms our initial observations and indicates that HSP90α functions to promote survival in differentiated cells in the presence of survival factors, but that constitutive expression sensitises these immortal mammary epithelial HC11 cells to starvation-induced apoptosis.

### HSP90α promotes survival in breast cancer cells

**Figure 7:**
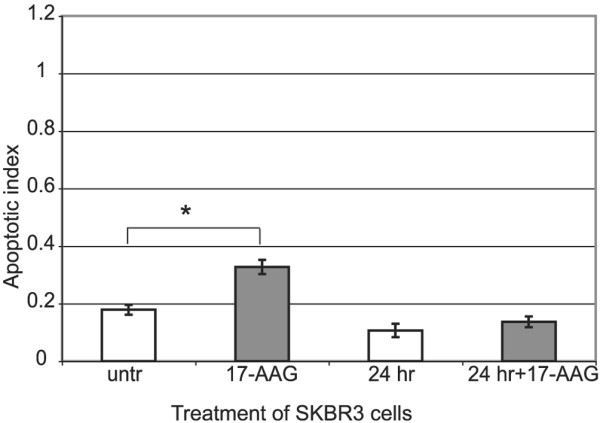
**HSP90α promotes survival only in the presence of serum in SKBR3 breast cancer cells**. Apoptosis was assessed by measuring the presence of mono- and oligo-nucleosomes as an indication of apoptosis (apoptotic index). Addition of 500 nM 17-allylamino-17-demethoxygeldanamycin (17-AAG) to SKBR3 cells increases apoptosis (t-test *p = 0.01). Starvation alone reduces the overall level of apoptosis observed in SKBR3 cells with serum (untr) (t-test p = 0.004). Each bar represents the average of three experiments with standard deviation. HSAP90α = heat shock protein 90 alpha.

Increased expression of HSP90α has been reported in breast cancer, including SKBR3 cells [35], and cytotoxicity has also been reported for the use of HSP90α inhibitors in breast cancer cells, including SKBR3 [68-70]. To test the role of HSP90α in SKBR3 cells, we assessed oligo-nucleasome formation in the presence and absence of 17-AAG and in the presence or absence of serum. HSP90α promotes survival, in the presence of serum, as indicated by the increase in apoptosis after treatment with 17-AAG (Figure 7). Interestingly, overall there was less apoptosis in the absence of serum and the amount of apoptosis was independent of 17-AAG. In general, cancer cells are known to be resistant to apoptotic stimuli, but the role of HSP90α in the absence of serum seems to be minimal. HSP90α promotes survival of SKBR3 cells in the presence of serum.

## Discussion

We used a subtractive hybridisation approach to identify a number of prolactin-regulated target genes in the human breast cancer cell line SKBR3. By focusing on the *HSP90A *gene in particular, we determined that the HSP90α protein has the potential to regulate survival differently in normal (immortal) mammary epithelial cells depending on the context of the hormonal milieu and the constitutive expression of the gene encoding HSP90α. As HSP90α is a central therapeutic target for breast cancer treatment, this also elevates the importance of prolactin and specifically identifies one mechanism for its contribution to breast cancer.

### Prolactin regulated genes in breast cancer cells

We identified a number of genes in our screen of breast cancer SKBR3 cells whose expression either increased or decreased in response to prolactin. Some of the genes have been previously associated with cancer progression (T complex protein-1 beta [71], tetratricopeptide repeat protein-4 [72]), cancer survival (Bax inhibitor 1 [73], mitofusion 2 [74,75]), heterogeneous nuclear riboprotein A1 [76]), drug resistance (T complex protein-1, HSP70 [77]), or cancer cell migration (Hs1 binding protein [78,79]). Prolactin signalling has been implicated in each of these phenomena in breast cancer cells though its role in drug resistance has not yet been thoroughly examined.

### Prolactin-JAK2-STAT5-regulates *HSP90A*, a therapeutically important gene

In this study, we also identified *HSP90A *as a prolactin-induced STAT5-activated target gene. HSP90α is a molecular chaperone of a large number of proteins involved in critical signal transduction pathways. The role of HSP90α downstream of prolactin helps explain the multiple effects of prolactin in normal cells and emphasises the significant contribution of prolactin-JAK2-STAT5 signal transduction to breast cancer.

HSP90 in cancer cells is present in an active form, in a multi-chaperone complex with high ATPase activity, in contrast to the HSP90 in normal cells, which is in an inactive, uncomplexed form. It is thought that these differences account for the high affinity of cancer-associated HSP90α for the inhibitory ATP mimetic drugs such as 17-AAG [38]. Chemotherapeutic drugs, such as 17-AAG, inhibit HSP90 and usually result in the degradation of HSP90 client proteins [80]. There are multiple client proteins of HSP90α, including steroid hormone receptors such as oestrogen receptor, protein kinases, cell cycle proteins and transcription factors that are essential targets in cancerous cell growth, survival, immortalisation, angiogenesis and metastasis [81]. The prolactin-mediated induction of HSP90α implicates prolactin in the acquisition or maintenance of each of those cancer-related traits.

### Prolactin is a survival factor in HC11 cells

We also determined that prolactin, dexamethasone and insulin, each act as survival factors in differentiated mammary epithelial HC11 cells. Insulin [66,82] and the glucocorticoid receptor have previously been identified as survival factors [83,84]. There is existing evidence showing a survival role for prolactin in breast cancer cells [59-63], and for STAT5 in normal mammary epithelial cells [64,65]. The survival function of prolactin is partially due to the prolactin-mediated activation of AKT/protein kinase B [85,86]. AKT/protein kinase B is a survival factor and a regulator of mammary gland involution, as transgenic mice expressing constitutively active AKT/protein kinase B in the mammary gland showed delayed involution and a delayed onset of apoptosis [87,88]. AKT/protein kinase B is also a client protein of HSP90 [89,90].

### HSP90α promotes survival depending on the cellular context

The prolactin-JAK2-STAT5 target gene, HSP90α, can contribute to survival in the presence of prolactin, but sensitises untransformed mammary epithelial HC11 cells to starvation-induced apoptosis when constitutively expressed. We confirmed these results with the use of 17-AAG.

There is evidence for the pro-apoptotic function of HSP90α in other cell types [91-93]. We can also hypothesise that the pro-apoptotic function is due to the action of one of the client proteins either stabilised under these conditions, such as mutant p53, or disengagement from one of its client proteins such as AKT/protein kinase B. Although it is known that HSP90 stabilises mutant p53 forms [94], many of these mutant forms contribute to cellular immortalisation and transformation. The mutant forms of p53 in HC11 cells are thought to contribute to their immortalisation [95], but it is not known if, under certain conditions, mutant p53 could contribute to the sensitisation of cells to starvation-induced apoptosis as does wild-type p53 [96]. The fact that HSP90 can stabilise mutant p53 forms that can contribute to immortalisation and transformation emphasises a contribution of prolactin to these functions, as one of its upstream inducers.

We propose that the switch from survival to apoptosis in untransformed cells involves the loss of survival factors and the availability of HSP90α. In contrast, the switch is absent in SKBR3 breast cancer cells, which do not respond to 17-AAG during serum starvation. HSP90 is important for proliferation and survival, as SKBR3 cells have been shown to respond to 17-AAG by a reduction in proliferation [68,70] and an increase in apoptosis [68,69]. This latter observation is consistent with our results in this report. Possible mechanisms involved in loss of HSP90-mediated survival after 17-AAG treatment include the loss of AKT/protein kinase B [97] or ERBB2 [69,98,99].

Together with our results, this indicates that in addition to a role in survival, HSP90α also has a pro-apoptotic role that may be cell-type specific, specific to the hormone milieu in the environment or specific to the cellular state of transformation and complement of tumour-suppressor proteins. We hypothesise that HSP90α together with prolactin-mediated events support survival in differentiated or cancerous cells, whereas HSP90α alone may sensitise differentiated mammary cells to wild-type p53-independent apoptosis depending on the cellular context.

## Conclusion

The evidence for a contribution of prolactin and STAT5 to breast cancer cell survival, breast cancer progression and to chemotherapeutic response is strengthened by our observations that prolactin treatment of human breast cancer cells regulates a number of genes associated with cancer progression, including the therapeutically important target gene, *HSP90A*. HSP90α is important for malignant progression in breast cancer, but when elevated in untransformed mammary epithelial cells may participate in a switch between survival and apoptosis.

## Abbreviations

17-AAG: 17-allylamino-17-demethoxygeldanamycin; bp: base pairs; *CIS*: cytokine inducible SH2 containing protein; EGF: epidermal growth factor; ERBB2: erythroblastic leukaemia viral oncogene homolog 2; HRP: horseradish peroxidase; HSP90: heat shock protein 90; JAK2: Janus kinase-2; STAT: signal transducers and activators of transcription.

## Competing interests

The authors declare that they have no competing interests.

## Authors' contributions

CP provided data regarding HSP90α protein levels. RL also participated in characterising HSP90α levels and the response of cells to prolactin. CTP helped with luciferase assays. NB performed EMSA and northern blots. JS and PB provided bioinformatics support. CSS prepared and screened the library, contributing luciferase and apoptosis assays. EP, CSS and BG contributed to early project design. BG and CSS provided funding. CSS provided further project development and wrote the manuscript. All authors read and approved the final manuscript.
